# Role of early experience in ant enslavement: a comparative analysis of a host and a non-host species

**DOI:** 10.1186/1742-9994-2-13

**Published:** 2005-08-02

**Authors:** Rumsaïs Blatrix, Claire Sermage

**Affiliations:** 1 Laboratoire d'Ethologie Expérimentale et Comparée CNRS UMR 7153, Université Paris 13, 99 av. JB Clément 93430 Villetaneuse, France

## Abstract

**Background:**

Ants use the odour of the colony to discriminate nestmates. In some species, this odour is learned during the first days following emergence, and thus early experience has a strong influence on nestmate discrimination. Slave-making ants are social parasites that capture brood of other ant species to increase the worker force of their colony. After emerging in the slave-maker nest, slave workers work as if they were in their own colony. We tested the hypothesis that early experience allows the deception of commonly enslaved species, while non-host species use a different mechanism, which does not involve learning.

**Results:**

Pupae of a host species, *Temnothorax unifasciatus*, and a non-host species, *T. parvulus*, were allowed to emerge in the presence of workers of one of two slave-maker species, *Chalepoxenus muellerianus *or *Myrmoxenus ravouxi*. When *T. unifasciatus *was exposed to slave-makers for 10 days following emergence, they were more aggressive towards their own sisters and groomed the slave-maker more. *T. parvulus *gave a less clear result: while workers behaved more aggressively towards their sisters when exposed early to *C. muellerianus *workers, this was not the case when exposed early to *M. ravouxi *workers. Moreover, *T. parvulus *workers allogroomed conspecific nestmates less than *T. unifasciatus*. Allogrooming activity might be very important for the slave-makers because they are tended by their slaves.

**Conclusion:**

Our findings show that early experience influences nestmate discrimination in the ant *T. unifasciatus *and can account for the successful enslavement of this species. However, the non-host species *T. parvulus *is less influenced by the early environment. This might help to explain why this species is never used by social parasites.

## Background

The early stages of an animal's life constitute a critical period during which the environment can have dramatic and sometimes irreversible effects on adult behaviour. The best example is imprinting, a process by which the individual develops an irreversible reference pattern from a stimulus present during a short period of time during its development. Lorenz [1] was the first to conceptualize the imprinting process and gave one of the most striking examples: during the few minutes after hatching, young birds developed an attachment to their parents if present, or a parental surrogate, such as Lorenz himself. Imprinting is only a special case of the processes by which early experience affects subsequent adult behaviour. For instance, in monkeys and humans, Harlow & Harlow [2] and Bowlby [3] noted the importance of attachment during the early phase after birth for the development of adult behaviour.

Despite a much simpler brain structure, insects display similarly pronounced plastic responses to the early environment. Influences of the preimaginal environment on adult behaviour have been demonstrated as early as the late 1930's in *Drosophila *[4], and were later documented and shown to be involved in various contexts in many other species (see review in [5]). These contexts can have a social dimension in social insects. Some ants, for example, learn the colony odour during the early phase of imaginal life and later use this template for recognizing brood [6-11] and discriminating among adults [12-15]. This critical period determines who benefits from altruistic behaviours shown by worker ants, which rarely reproduce directly but rely on indirect fitness benefits via reproduction of related individuals [16].

Slave-making ants are social parasites that exploit the labour force of other ant species. Slave-maker workers are specialized for conducting raids, wherein they seize brood from nearby host ant colonies and bring them back to their own nest [17]. When they emerge, the slave ants behave as if they were in their own colony. Among other routine ant tasks, they rear the slave-maker brood, defend the nest, and sometimes feed and groom the slave-maker workers. Altruistic acts of slaves are thus directed toward unrelated individuals. One hypothesis suggests that slave deception is possible because slaves are captured as pupae and learn the slave-maker colony odour after emergence [18-20].

*Myrmoxenus ravouxi *and *Chalepoxenus muellerianus *are among the most common slave-makers in Western Europe. They use several related species as slaves but show a preference for *Temnothorax unifasciatus *[21]. In the Mediterranean area, *M. ravouxi*, *C. muellerianus *and *T. unifasciatus *can be found in sympatry with *T. parvulus*, though *T. unifasciatus *seems to prefer more arid environments. *T. parvulus *has never been found as a slave, but the reason for this is unknown. We postulated that slavery would be prevented if nestmate discrimination in *T. parvulus *was not achieved through early learning of colony odour, because slave workers emerging in the slave-maker colony would recognize the slave-maker brood and workers as aliens.

Our study had two aims. First, we tested the hypothesis that deception of the host *T. unifasciatus *ensues from early learning of colony odour as a mechanism for nestmate discrimination. Second, we tested the hypothesis that early experience (within the first 10 days following emergence) affects the discrimination capabilities of *T. parvulus *workers less than those of *T. unifasciatus*. After manipulating the early experience of *T. unifasciatus *and *T. parvulus*, we tested and compared their discrimination capabilities.

## Results

*T. unifasciatus *workers reared with *M. ravouxi *(batch A) or *C. muellerianus *(batch B) were significantly more aggressive towards their unfamiliar sisters than towards the familiar *M. ravouxi *or *C. muellerianus *workers (Table 1). Moreover they spent significantly more time allogrooming the familiar *M. ravouxi *or *C. muellerianus *workers than their unfamiliar sisters. Similarly, *T. unifasciatus *workers reared with conspecifics (batch C) were less aggressive towards their familiar sisters than towards the unfamiliar *M. ravouxi *and *C. muellerianus *workers. They also spent significantly less time allogrooming the unfamiliar *M. ravouxi *or *C. muellerianus *workers than the familiar sisters (Table 1).

**Table 1:** Discrimination capabilities of two ant species after manipulation of their early experience. Number of agonistic acts and duration (in seconds) of allogrooming behaviours of *T. unifasciatus *and *T. parvulus *workers tested during 10 minutes towards a *M. ravouxi*, *C. muellerianus *or a homospecific worker. Test workers were reared for 10 days after emergence either in the presence of *M. ravouxi *(batch A), *C. muellerianus *(batch B) or nestmate workers (batch C).

species tested	treatment (N,C)*	species presented	aggression (mean ± SE)	Z	P	grooming (mean ± SE)	Z	p
*T. unifasciatus*	batch A (22,10)	*M. ravouxi*	0.3 ± 0.2			16.5 ± 5.9		
				3.2	<0.002		3.2	<0.002
		*T. unifasciatus*	14.3 ± 6.4			0.4 ± 0.4		
	
	batch B (22,10)	*C. muellerianus*	0.8 ± 0.5			30.8 ± 9.5		
				3.5	<0.001		3.4	<0.001
		*T. unifasciatus*	14 ± 4			0.03 ± 0.03		
	
	batch C (22,10)	*M. ravouxi*	6.3 ± 3.2			0.9 ± 0.9		
				2.3	0.02		2.4	0.019
		*T. unifasciatus*	0.5 ± 0.3			19.3 ± 13.4		
				2	0.047		2.1	0.033
		*C. muellerianus*	3.2 ± 1.4			2.5 ± 2.3		

*T. parvulus*	batch A (22,5)	*M. ravouxi*	2.8 ± 1.6			2.7 ± 1.9		
				0.4	0.69		1.6	0.11
		*T. parvulus*	2 ± 0.8			0 ± 0		
	
	batch B (23,8)	*C. muellerianus*	1.9 ± 0.7			4.9 ± 3.6		
				2.3	0.02		2.4	0.018
		*T. parvulus*	4.6 ± 1.4			0 ± 0		
	
	batch C (22,8)	*M. ravouxi*	3.4 ± 1.5			3.5 ± 2.4		
				2	0.049		0.7	0.47
		*T. parvulus*	0.2 ± 0.1			0.1 ± 0.1		
				2.5	0.013		1.3	0.50
		*C. muellerianus*	3.2 ± 1.8			0 ± 0		

* N: number of ants tested; C: number of colonies the test ants originated from.

*T. parvulus *workers reared with *M. ravouxi *(batch A) did not show a significant difference in aggressive behaviour or in the time spent allogrooming between their unfamiliar sisters and the familiar *M. ravouxi *workers (Table 1). Conversely, *T. parvulus *workers reared with *C. muellerianus *(batch B) were significantly more aggressive towards their unfamiliar sisters than towards the familiar *C. muellerianus *workers, and they spent significantly more time allogrooming the familiar *C. muellerianus *workers than the unfamiliar sisters. *T. parvulus *workers reared with conspecifics (batch C) were significantly less aggressive towards their familiar sisters than towards the unfamiliar *M. ravouxi *or *C. muellerianus *workers, but the time they spent allogrooming the unfamiliar *M. ravouxi *or *C. muellerianus *workers and their familiar sisters was much reduced and did not differ significantly (Table 1).

*T. unifasciatus *and *T. parvulus *workers reared with conspecifics (batch C) did not show any significant difference in the number of aggressive acts towards *M. ravouxi *(6.3 ± 3.2 and 3.4 ± 1.5 respectively, Z = 1.2, p = 0.22) or *C. muellerianus *(3.2 ± 1.4 and 3.2 ± 1.8 respectively, Z = 0, p = 0.99). However, *T. unifasciatus *workers reared with conspecifics (batch C) spent significantly more time allogrooming familiar sisters than did *T. parvulus *(19.3 ± 13.4 and 0.1 ± 0.1 respectively, Z = 2.6, p = 0.01).

## Discussion

Our results suggest that the environment experienced by *T. unifasciatus *during the first 10 days after emergence influences nestmate discrimination, at least in our artificial laboratory conditions. Indeed, workers that were exposed early to *M. ravouxi *and *C. muellerianus *displayed a very aggressive behaviour towards their own sisters and spent much time grooming the slave-makers. This suggests that the influence of the early environment could potentially account for the fact that *T. unifasciatus *slaves care for and defend the slave-makers. However, as host workers never emerge only with slave-makers present the mechanisms allowing enslavement could be different in nature. The same result was obtained for *T. parvulus *workers that were exposed to *C. muellerianus*. However, when exposed to *M. ravouxi*, they were not more aggressive toward their sisters than toward the slave-maker. These results partly confirm our hypothesis that the effect of early experience on the discrimination capabilities is reduced in *T. parvulus *when compared with *T. unifasciatus *workers.

The use of tethered, but live ants to record the reaction of test workers could have influenced their behaviour through the performance of antennation, biting, stridulation, etc., and the emission of pheromones. Indeed, several species of social parasites are known to use repellent, appeasement, and/or propaganda substances to usurp host nests [22]. The occurrence of such substances in our focal slave-maker species is likely and could have reduced aggressiveness of test workers. Moreover, several pupae of the test species were allowed to emerge in the glass tubes containing slave-makers. The early experience of test workers could then have been influenced by the other emerging test workers. Another potential source of bias in our results is the unequal representation of collected populations of *Temnothorax *in the experimental groups. The observed differences between our *T. parvulus *and *T. unifasciatus *might be due to differences between populations rather than species, if early experience had a smaller influence on nestmate discrimination in the Italian population. However, this possibility seems unlikely because species differences for fundamental mechanisms (such as the determinant of nestmate discrimination) are expected to be unaffected by population differences. The observed differences between the two species cannot be due to a mere difference in aggressiveness, because their levels of aggression towards slave-makers in the control experiment were not significantly different. However the difference between their levels of allogrooming was highly significant. A low basic level of allogrooming activity for *T. parvulus *workers was probably responsible for the fact that they did not groom their sisters more than the slave-makers in the control experiment. This makes it difficult to test the influence of early experience on allogrooming behaviour of *T. parvulus*.

Few other studies have demonstrated that early experience with slave-maker workers or brood elicits slave-maker care in the host species [23-25]. These studies dealt with species in the subfamily Formicinae; specifically, the slave-maker *Formica sanguinea *and its hosts *F. fusca *and *F. cunicularia*. Slave-making ants are known from only two subfamilies: the Formicinae and the Myrmicinae [26]. In the only study considering Myrmicinae, Alloway and Hare [27] showed that early learning was not necessary to explain the acceptance of the brood of the slave-maker *Protomognathus americanus *by enslaved *T. longispinosus *workers. Indeed, slave-maker larvae were preferentially accepted even when *T. longispinosus *workers were exposed early to conspecific larvae. Our results confirm that early experience can be important for successful ant enslavement in other myrmicine systems. Together, these studies across subfamilies seem to confirm the hypothesis that early behavioural plasticity of certain ant species has permitted or at least facilitated the evolution of slave-making habits [28], and show that other mechanisms are possible [27] but seem less common. This suggests that early behavioural plasticity could be a general prerequisite for the evolution of slave-making in ants [20]. The fact that early experience is important for the integration of the young slaves into the slave-maker colony might explain why *M. ravouxi *and *C. muellerianus *do not capture adult hosts during raids [29,30]. In fact, out of 10 genera displaying interspecific slavery, only one is known to result in the mixing of adult slaves into the slave-maker colony [26]: raids of the slave-making *Strongylognathus *commonly end in the fusion with the *Tetramorium *host nest [31]. However, the fusion occurs after a prolonged fight that often results in casualties on both sides. It is noteworthy that intraspecific colony fusion has been reported in the genus *Tetramorium *as a result of territorial competition [26].

For some slave-making species, host colony take-over by slave-maker queens requires acceptance by adult host workers that have had no previous exposure to slave-makers. The founding queen of many slave-making species has to enter a host nest, be accepted by the host workers, then kill and replace the resident queen [19]. Once the slave-making queen manages to kill the resident queen, the workers care for the new queen and rear her brood. The acceptance of the new queen does not require previous exposure to slave-makers because specialised behavioural and chemical strategies are involved [22]. For example, in the genus *Polyergus*, the young slave-maker queen does not bear any odour, and thus prevents the host workers from detecting her. The slave-maker queen then acquires the cuticular hydrocarbons of the host queen by physical contact and the host workers accept her as though she were there natal queen [32,33]. The founding queen of *M. ravouxi *enters the *Temnothorax *host nest, reaches the queen, and slowly throttles her to death [34]. The chemical mechanism responsible for the acceptation of the *M. ravouxi *queen by the host workers remains to be elucidated.

Queens of other slave-making species including *C. muellerianus*, *Harpagoxenus *and *Protomognathus *evict all adult ants from the host nest and keep only the host brood. They thus rely completely on the manipulation of early experience even during colony foundation. The life history of slave-making ants suggests that the use of adult slaves without previous exposure to the slave-maker is costly or involves a highly specialised strategy. Manipulation of early experience by the slave-maker appears as the most parsimonious strategy, which reinforces the suggestion that it facilitated the evolution of slavery.

Early behavioural plasticity is a widespread mechanism in animals and is involved in a number of host-parasite systems. However, in most cases, this mechanism is used by the parasite to find its host. The European cuckoo is known to rely on habitat, probably in addition to other cues, which is learned when reared by the host [35], but it does not seem to imprint directly on the host [36]. In parasitoid wasps, preimaginal imprinting is involved in host selection [5]. In slave-making ants, imprinting was also shown to influence host selection during raids and colony foundation [37,38].

A striking result of this study was that manipulating the early experience of *T. parvulus *had different consequences depending on the slave-maker species to which it was exposed; nestmate discrimination seemed to be influenced by *C. muellerianus*, but not by *M. ravouxi*. If *T. parvulus *was insensitive to experience at emergence due to a strict genetic system of odour discrimination or an earlier sensitive period, we would expect the same outcome for both slave-maker species. A possible explanation is that there is a limited set of odours that *T. parvulus *can learn and/or perceive at emergence. *M. ravouxi *and *C. muellerianus *likely have different chemical profiles and *T. parvulus *might be chemically more similar to *C. muellerianus *than to *M. ravouxi*. Thus, the latter could be out of the range of potentially learned patterns. As we show in our experiments, phylogenetically closely related ant species can display various sensitivities to the early environment. Thus, perhaps the mechanisms of nestmate discrimination differ as well.

Nestmate discrimination has been shown to be less influenced by social environment at emergence in the ant genus *Camponotus *than in the genus *Formica *[7]. It is therefore interesting that no species of *Camponotus *is parasitized by slave-makers, while many *Formica *species are hosts to slave-makers. Similarly, our results on *T. parvulus*, the non-slave species, suggest that plasticity at emergence is not the only determinant of nestmate discrimination. A first alternative possibility is that nestmate discrimination is determined during an earlier sensitive period. Indeed, studies in *Camponotus floridanus *and *Cataglyphis cursor *demonstrated the importance of larval stages for nestmate discrimination [39,40]. As most slave-makers capture pupae preferentially, a nestmate discrimination mechanism based on larval experience might prevent these species from being enslaved. A second possibility is that nestmate discrimination in *T. parvulus *has a genetic component or is based on self-referent phenotype matching. *T. nylanderi*, a species very closely related to *T. parvulus*, has nestmate discrimination cues based on colony environment, and especially nest site material [41]. Even if *T. parvulus *can be expected to rely in part on a similar mechanism in the field, it could not have influenced our results because colonies were reared in identical artificial nests. Whatever the determinant of nestmate discrimination in this species, it can be an obstacle to slavery because at least some elements of the template involved in discrimination are established before the slaves are captured. Moreover, *T. parvulus *workers do not groom their nestmates often, which could be problematic for the slave-makers because they are tended by the slaves. These characteristics might partly explain why *T. parvulus *has never been found as slave. However, other factors could explain why *M. ravouxi *and *C. muellerianus *does not enslave *T. parvulus*. Social parasites in the tribe Formicoxenini are phylogenetically closely-related to their hosts [42]. It might be impossible for the social parasites to exploit more distant species as hosts because they would not share the same ecological and microhabitat requirements, or the same communication system. Slave-maker species could also be under selection to match the colony odour of the host species. Coevolution occurs between slave-making ants and their hosts [43,44] and it has been shown that in slave-maker colonies both species have similar cuticular hydrocarbon profiles [45,46]. It might therefore be easier for the host species to learn the more similar profile of the parasite, than for the non-host species.

## Conclusion

Early experience can have important consequences on the behaviour of adult ant workers and consequently on their inclusive fitness. Manipulation of early experience in an experimental (this study) or natural (social parasitism) context influences nestmate discrimination. However, we showed that the effect varies across species: it was more pronounced in the host than in the non-host species. Social parasitism is not evenly distributed in ants: most of the 200 known species occur in only two subfamilies (the Formicinae and the Myrmicinae), and in these subfamilies it is concentrated in a few genera [47]. The reasons for this distribution are still unknown and elucidating the fine scale mechanisms involved in nestmate discrimination across taxa might help to explain the evolution of slavery in ants.

## Methods

Colonies of the two slave-maker species and of both host species were collected in northern Italy (Lago di Garda) and southern France (Vaison-la-Romaine and Grasse, we found no slave-makers near Grasse) in the spring and summer 2003. Because of a sampling bias, significantly more *T. unifasciatus *originated from southern France and more *T. parvulus *from northern Italy (Chi-square = 13, p = 0.022). Nests were housed in plastic boxes containing a thick layer of plaster. The print of a microscope slide covered by a glass plate provided a nest site [48]. Colonies were fed twice a week with frozen fruit flies and a mixture of honey and apple. Rearing conditions were as follows: day/night: 14 h-24°C/10 h-17°C. Genus names comply with Bolton's new classification of Formicidae [49]. The experiments were performed in August and September 2003.

Pupae of *T. unifasciatus *and *T. parvulus *that were close to emergence were removed from their colonies (unparasitized) and isolated in glass tubes with either *M. ravouxi *(experimental batch A), *C. muellerianus *(experimental batch B) or homo-colonial (control batch C) workers. Each glass tube contained one to three pupae (from the same colony) and two to six adult workers. The small numbers of slave-maker workers available precluded larger worker/pupae ratios, but at least two adult workers were used for each pupa. Slave-maker workers of *M. ravouxi *and *C. muellerianus *were obtained from 20 and 27 colonies respectively. Pupae close to emergence were recognizable by pigmentation, and emergence took place within two days of uniting pupae with their respective conditioning workers. Amputation of the distal segments of one leg of adult *Temnothorax *workers from batch C allowed us to distinguish them unambiguously from the newly emerged test workers. Slave-makers from batches A and B were not subjected to this amputation. Workers were tested 10 ± 0.3 (mean ± SE) days after emergence.

Each test worker was introduced into a circular arena of 1.5 cm^2 ^where another worker was tethered by a nylon thread tied between the head and alitrunk. We were only interested in the behaviour of the test worker, thus we tethered the other worker to reduce the probability of interaction. Tethered workers could, however, still emit pheromones and engage in various other behaviours such as antennation, biting, stridulation etc. Tests started at least five minutes after tethering in order to let the tethered worker acclimate, but we cannot totally exclude the influence of pheromones on the behaviour of test workers. Duration of allogrooming and number of agonistic acts (mandibles opening, biting, stinging attempts) towards the tethered worker were recorded over 10 minutes (from the first encounter). Each test worker from batches A and B was tested successively against a slave-maker worker (*M. ravouxi *or *C. muellerianus *respectively) from its glass tube (hetero-colonial and familiar) and a worker from its colony of origin (homo-colonial and unfamiliar). Test order was controlled. Duration of allogrooming and number of agonistic acts were compared between the two tests using the Wilcoxon test for paired samples. Each test worker from batch C was tested successively against a worker from its glass tube (homo-colonial and familiar), a *M. ravouxi *worker, and a *C. muellerianus *worker (hetero-colonial, unfamiliar). All combinations of test order were equally represented. The test involving the homo-colonial and familiar worker was compared to each of the two tests involving a slave-maker worker using the Wilcoxon test for paired samples. Moreover, the number of agonistic acts towards the slave-makers was compared between *T. unifasciatus *and *T. parvulus *with a Mann-Whitney test for independent samples in order to test for a difference in levels of aggression between the two species. Differences in duration of allogrooming between the two species were similarly tested by comparing the performances of test workers from batch C in tests involving a homo-colonial and familiar worker. Tests were computed with Statistica 6.

## Authors' contributions

RB conceived the study, carried out part of the experiments and data analysis, and drafted the manuscript. CS participated in the design of the study, carried out part of the experiments and data analysis. Both authors read and approved the final manuscript.
